# An Investigation on Pervasive Technologies for IoT-based Thermal Monitoring

**DOI:** 10.3390/s19030663

**Published:** 2019-02-06

**Authors:** Edoardo Giusto, Filippo Gandino, Michele Luigi Greco, Michelangelo Grosso, Bartolomeo Montrucchio, Salvatore Rinaudo

**Affiliations:** 1 DAUIN, Politecnico di Torino, Corso Duca degli Abruzzi, 24, 10129 Torino, Italy; filippo.gandino@polito.it (F.G.); micheleluigi.greco@studenti.polito.it (M.L.G.); bartolomeo.montrucchio@polito.it (B.M.); 2 STMicroelectronics s.r.l., Corso Duca degli Abruzzi 24, 10129 Torino, Italy; michelangelo.grosso@st.com; 3 STMicroelectronics s.r.l., Via Franco Gorgone 39/41, 95121 Catania, Italy; salvatore.rinaudo@st.com

**Keywords:** sensors, RFID, bluetooth, thermal monitoring

## Abstract

Indoor thermal monitoring is a crucial requirement for home automation, which fits inside the ever-growing Internet of Things (IoT) paradigm. The IoT ecosystem aims at connecting every device exploiting specific functions, deployed in a particular place, in order to give the chance to the users to monitor and/or control some aspects of their life, or to demand this task to a proper software. In the thermal monitoring context, IoT provides new opportunities for a dense and/or large-scale distribution of sensors, which have to gather data in order to effectively control the Heating, Ventilation and Air Conditioning (HVAC) system. Several wireless technologies can be exploited for this scope. However, they involve different benefits and drawbacks. In particular, this study is focused on Radio Frequency Identification (RFID) and Bluetooth®, which represent two well-known wireless technological standards used by commercial electronics but suitable also for pervasive IoT systems. These technologies are discussed and compared from several points of view, i.e., flexibility, reliability, battery life and cost of the system. A theoretical analysis highlights their benefits for the application context and evaluates their suitability to dense and large-scale monitoring systems. The theoretical results are supported by an experimental analysis based on the implementation and test of two different systems, one using RFID and the other using Bluetooth technology.

## 1. Introduction

Computer Science and Engineering have evolved in time, from a stand-alone server or desktop to an embedded form. Embedded computing is pervasive, yet usually not seen. Embedded computers, sensors and actuators are put together to form the Internet of Things (IoT) [1] framework. Systems working in this domain are designed to be not only completely autonomous, but are going to provide useful or necessary services to human beings, such as the chance to analyze some data in real time and take actions or decisions based on them.

IoT-based applications are currently studied and exploited in many sectors, such as health-care [2,3], autonomous vehicles [4], and environmental monitoring (e.g., air [5], water [6] and fire monitoring [7]). Home automation is the field studying the exploitation of technology for quality of life improvement inside buildings [8,9].

The smart environment paradigm can be applied to various kinds of closed settings: private houses, offices, laboratories, factories etc. Automated systems can control many appliances inside a smart environment, for instance doors opening and locking. One of the most important aims is controlling the Heating, Ventilation and Air Conditioning system (HVAC), which is directly responsible for the wellness of the user inside the building. The aim of this kind of systems is to automatically control temperature (${}^{\circ }$C) and relative humidity (RH%) in order to maximize the comfort for the users while reducing as much as possible the energy consumption.

The American Society of Heating, Refrigerating and Air-Conditioning Engineers defined thermal comfort as “the condition of mind that expresses satisfaction with the thermal environment and is assessed by subjective evaluation” [10].

To automatize the HVAC process, sensors are needed to numerically measure the environmental conditions, while actuators are needed to perform the required actions. A fundamental block of this process is the measurement of temperature and relative humidity. A related issue is the deployment density of sensors, which depends on the specific environment and on the granularity of the HVAC system. The actual configuration of the environment has to be carefully taken into account when determining the deployment locations of the sensors. For sure, the denser is the deployment, the higher is the accuracy of the measurement, but redundancy could be avoided. Wireless sensors can be leveraged so as to effectively build a pervasive distributed monitoring system. Traditionally WSNs are composed of low-cost devices, featuring: a low-frequency microcontroller, a RAM memory comprised between 2 kB and 32 kB, a limited capability to no Operating System. The majority of these devices use IEEE 802.15.4 standard for communication [11]. Buildings-oriented WSNs should fulfill a series of characteristics [12]: high correct transmission rate, low power requirements, reconfigurability, and scalability.

Although many papers consider the use of thermal monitoring systems, high-density wireless implementations are still an emerging application and there do not exist stable guidelines about the best enabling technologies. This paper investigates the use of widespread communication systems for commercial electronics applied to the task of thermal monitoring. Radio Frequency IDentification (RFID) and Bluetooth technologies have been selected even if not traditionally strictly applied to WSNs. These technologies are both widespread, cheap, low power and already used in similar applications.

The RFID technology was originally designed to discern among many instances of the same object in a quick way using wireless communication. As technology scaled, it has been possible to integrate sensors directly in the system hardware and this led to the growing use for various applications in different fields: smart buildings [13] access control [14], supply chain traceability [15], and healthcare [16]. RFID transponders are small, cheap and low-power consuming. Moreover, the presence of RFID tags integrated with sensors makes RFID a proper candidate for dense thermal monitoring.

The Bluetooth® technology aims at providing an inexpensive, low-power, short-range radio-based interface between devices of different nature. It is usually used to develop a Personal Area Network (PAN), where a number of slave devices are connected to a master. From the first specifications by the Bluetooth Special Interest Group (SIG) formalized in 1998, many improvements have then been provided. In 2010, the 4.0 protocol version introduced Bluetooth Smart, defining Classic Bluetooth, Bluetooth High Speed and Bluetooth Low Energy (BLE). BLE is a subset of Bluetooth v4.0 with an entirely new protocol stack for rapid build-up of simple links. The latest specifications have reached version 5, presented in June 2016. Bluetooth is largely used in IoT applications. It provides a good communication range and requires low-power modules that can be integrated into embedded devices, as required by a dense monitoring system. In order to evaluate the possible benefits for thermal monitoring applications and to evaluate the applicability to dense and large scale systems, RFID and Bluetooth are carefully analyzed and compared.

The analysis covers many aspects: flexibility, reliability, battery life and cost of the system. Moreover, two prototype implementations of a thermal monitoring system have been done by using RFID and Bluetooth, respectively. The implementations have been tested in order to validate the results of the theoretical analysis. Each application location involves specific issues and requirements. Therefore, the purpose of the performed tests is not to provide the exact performance of the considered technologies, but to observe their general behavior in a real scenario and suitability for a specific HVAC application. A preliminary version of the analysis on RFID for thermal monitoring was published in [17]. The current paper extends the original work with an accurate theoretical analysis, with a theoretical and experimental investigation on Bluetooth systems, and with a comparative analysis.

The rest of the paper is organized as follows. In Section 2, related works are described. Section 3 presents the theoretical analysis and comparison, while in Section 4 a practical comparison is carried out. Section 5 presents the system implementation and describes the performed tests. Finally, conclusions are drawn in Section 6.

## 2. Related Works

Two main approaches exist for the task of thermal monitoring: wired or wireless. The wireless one is often preferred, since the tradeoff between using wireless devices and wiring a particular environment favours the former case.

Wireless sensor networks (WSNs) are made up by several (generally) low-cost devices, able to collect certain data related to the environment and to send these data via wireless communication. Wireless devices operating in this framework take the name of *sensor nodes*.

Applications of WSN nodes to HVAC monitoring and control systems already exist. In [18], an algorithm is proposed to forecast the temperature evolution of an indoor environment exploiting data coming from wireless nodes. An artificial neural network was implemented using low-cost, low resources nodes, based on an on-line learning approach, without the use of a historical database, due to the limitation of the hardware and the system in general. Nevertheless, the presented model is really effective in forecasting the evolution of temperature even after a short period of time in an unknown environment. The nodes were produced by Wireless Sensor Networks Valencia SL, embedding a CC1110F32 Texas Instruments™ microcontroller.

A key issue in this domain is related to the power supply mechanism of the nodes. If they are supplied by a battery, this could need replacement or recharging connected to an electric socket. To avoid this hassle, a self-powered wireless node has been proposed in [19], applied to HVAC monitoring task. These kinds of devices are shown able to easily integrate with pre-existent energy saving systems, also supporting diagnostics via dedicated alerts, all this requiring almost no intervention at all from the users.

WSNs are also leveraged for more than a single purpose at a time. In [20], the main task is the monitoring of indoor air quality, but the network is not limited to the use of air quality sensors. Instead, also pervasive data about temperature are collected at the same time. In this work, the devices used as sensing nodes were the widespread Waspmotes, manufactured by Libelium™.

RFID technology is not commonly applied to the task of thermal monitoring for HVAC systems. The original purpose of this technology was related to the object identification. Nevertheless, nowadays several examples exist of RFID tags also embedding temperature sensors, which are mainly used to track the cold chain of perishable goods. In [21] a general-purpose CMOS temperature sensor is proposed and carefully described. In [22] instead, a temperature sensor tag is proposed to be installed directly inside the concrete walls of buildings during construction.

In [23], a hybrid temperature monitoring system has been presented. RFID tags and WSN nodes are combined together to sense the temperature in a refrigerator chamber. Used tags are HF, semi-passive ones, with short communication range. WSN nodes are able to sample and transmit the data continuously, while the RFID tags are manually read by an operator.

RFID technology has been applied to HVAC automation in [24]. This study examined the impact of human presence detection inside a particular environment. Both stationary and mobile location of people were detected with fairly high precision. Such self-adaptive system is demonstrated to effectively reduce the energy consumption of a building.

Also, commercially available purely passive tags exist, for instance the ones manufactured by RFmicron™ (http://rfmicron.com/temp-sensor/) or Farsens™ (http://www.farsens.com/en/products/battery-free-rfid-sensors/temperature/). Since this kind of tags are battery-free, there is no way for them to unmannedly sample the environment in which they reside. The user has to manually query the tag every time a new data has to be collected.

Regarding Bluetooth®, there are not many scientific articles using such devices to sense temperature and relative humidity. There are instead several using Bluetooth® devices in HVAC systems to detect the occupation of rooms, such as [25,26]. The former is cheaper, leveraging the users’ mobile phones presence and Bluetooth® functionality, while the latter requires the installation of additional hardware for the purpose of effective triangulation of the signal to determine the position of the devices.

Not strictly related to indoor thermal monitoring, reference [27] proposes a multi-hop real-time communication protocol leveraging commercial BLE devices forming a mesh network for industrial applications. Such an approach could be implemented where there are strict requirements for what concerns the timing of the thermal monitoring task.

In [28] a forecast system for indoor temperature is presented. This has been achieved using sensors inside and outside an electrical appliance (refrigerator) and also outdoor temperature. The proposed solution is demonstrated to be very effective in predicting room temperature in order to reduce not only the power consumption of the electrical appliance itself, but also the data that can be used for finer tuning, exploiting the relation with the outdoor temperature. This could potentially lead to a decrease in power consumption in entire buildings or even in cities.

A framework for the development of indoor flexible temperature monitoring is presented in [29]. In this work, a mesh network is realized using off-the-shelf components, also implementing an aggressive synchronization scheme to minimize power consumption. The system has been effectively deployed in a real use case for a year-long continuous data acquisition campaign.

## 3. Technological Background

Both RFID and Bluetooth Low Energy solutions are characterized by a low energy consumption and a moderate data rate, usually up to a few hundred kbps. These characteristics make them suitable for the purpose of environmental monitoring. Just like every other wireless communication technology, RFID and Bluetooth® have to deal with possible interference. The denser is the communication medium, the more difficult the communication results. Furthermore, for what concerns RFID technology, the presence of metal in the medium or other radio communications, such as GSM [30], can interfere with their transmissions. In this section, a theoretical description of the two technologies will be performed.

### 3.1. RFID

An RFID system is made by one or more *tags* and one or more *receivers*. The reader/receiver queries tags via wireless communication. Tags are thus composed by a logic element, a memory element and a radio frequency antenna. It is possible to distinguish the kinds of tags depending on the different power supply mechanisms: *passive*, *semi-passive* and *active*. Passive tags do not have a battery, they are usually simply used for identification purposes (for example: tracking in the supply chain). They are powered only by the wireless communication of the reader. The electromagnetic radiation gives enough power to the tag so as to wake up and communicate the required information. Semi-passive tags include a battery to supply the sensors, while the communication with the reader is still achieved in a passive way. Active tags instead are equipped with a battery and are able to autonomously sample the sensors and ensure longer communication ranges with respect to passive ones. In addition, once properly programmed, they can autonomously initiate data sending.

RFID systems are subject to many interference issues. Water and metal can block the communications. Moreover, other RFID signals or other wireless transmissions operating at a close frequency can affect the communications. In particular, a reader can be prevented from receiving the low-power answers of passive tags. The mutual interference among RFID devices is called collision [31]. A tag-to-tag collision corresponds to two tags simultaneously answering to a reader. A reader-to-tag collision corresponds to a tag simultaneously queried by two readers. A reader-to-reader collision involves a reader that with its high power transmissions prevents another reader from receiving the answers of a tag. RFID tags are generally manufactured in simple and quite robust shape. Active tags are usually built with non-rechargeable batteries, thus the life expectancy of the device is directly related to its battery. In a network composed of this kind of tags, if one of them ceases functioning the system does not stop, thus this loss is not critical. Instead, the reader ceasing functioning actually stops the system and it has to be substituted. A recap of the various frequencies at which RFID systems work can be found in Table 1.

### 3.2. Bluetooth®

Bluetooth® is one of the main wireless technology standards operating over short distances. Bluetooth® operates at frequencies between 2402 and 2480 MHz, in the globally unlicensed Industrial Scientific and Medical (ISM) band. Data to be sent is divided into packets and then transmitted on one of 79 channels, each 1 MHz-wide. The channel is changed 1600 times per second according to the adaptive frequency hopping mechanism, used to overcome interference problems. Bluetooth Low Energy uses 2 MHz channel spacing, for a total of 40 channels.

The heart of the Bluetooth® specification is the Bluetooth® protocol stack. By providing well-defined layers of functionality, the Bluetooth specification ensures interoperability of Bluetooth devices and encourages adoption of Bluetooth technology. The layers range from low-level radio link to application and profiles. Lower layers handle packet management and physical data transmission; upper layers provide communication APIs to applications. The developed application is directly interfaced with the Generic Access Profile (GAP) APIs. GAP is a top layer in the host protocol stack that defines how BLE devices behave in standby and connecting states to maintain interoperability with peer devices. GAP also describes discovery, link establishment and security procedures.

**Figure 1:**
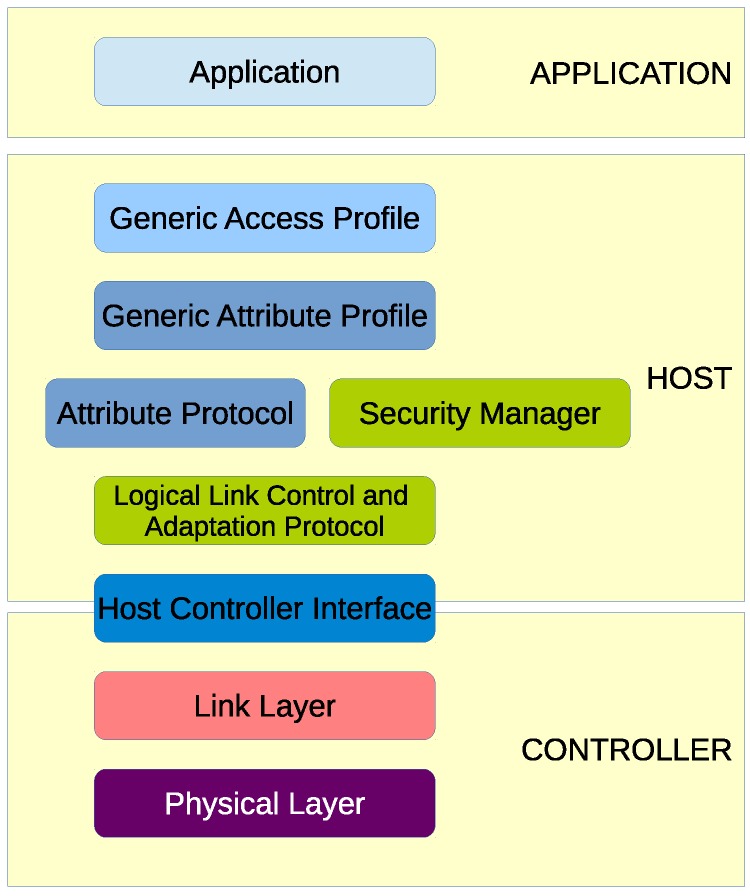
The Generic Access Profile (GAP) layer within the BLE protocol stack.

Figure 1 visually shows the GAP layer in relation to other layers in the software hierarchy.

BLE standard specifies GAP roles as shown in Table 2.

Bluetooth® devices send advertising packets (PDUs) to broadcast data on one or more channels, and to allow other devices (scanners) to find and connect to them. The advertising data consists up to 31 bytes of user configurable data. An additional 31 bytes can be sent as a scan response to a scan request.

For what concerns batteries powering Bluetooth® devices, there is no such mainstream approach between rechargeable/non-rechargeable or interchangeable/non-interchangeable. Different requirements for a special application could result in a different trade-off on these features. Taking into account the robustness of the overall system, it greatly depends on the network architecture. The single node ceasing functioning could have relative importance on the operation of the overall system. If a star network is chosen, a critical situation happens in case the sink node fails. Instead, if a mesh approach is in use, the failure of gateway or hop nodes could be compensated with a reconfiguration of the network.

## 4. Comparative Analysis

There are a few but critical requirements for the deployment of a thermal monitoring system: transmission range, scalability, power consumption, interference resilience and costs of the system. This subsection will focus on the characteristics of the system concerning these requirements, highlighting the pros and cons for the adoption of one of the two systems. In Table 3 a qualitative evaluation of the addressed features is presented.

### 4.1. Transmission Range

Typically, active RFID systems operate on a range of 100 m in open air environments. The network organization strictly follows the star distribution paradigm, with no possibility of network reconfiguration. For what concerns BLE devices instead, the point-to-point transmission range is more than 100 m, depending on the transmission power.

### 4.2. Scalability

The concept of scalability concerns both the number of devices in the system and the area it has to cover. Cover the area with RFID devices would mean to add in the system more readers, with the overhead in terms of hardware cost for the additional readers. In case the packet sent by a tag is read by more than one reader, the software should simply discard redundant information, which means to map every tag with one single reader within its range. For what concerns Bluetooth®, a similar approach can be used. In addition, this kind of devices could be programmed to create a mesh network. In this case, the multi-hop communication removes the need for additional readers.

### 4.3. Interference Resilience

As previously said, the RFID technology is actually subject to several kinds of interference, which are not avoidable due to the definition of the protocol. The collisions that affect RFID are effectively managed by specific protocols. The most common is the tag-to-tag collision. However, there are many protocols that allow identifying in a short time all the tags [32,33]. Reader-to-tags and reader-to-reader collisions are a problem only within environments with many readers. Also, this issue can be managed by existing protocols [34,35]. However, a degradation of the transmission efficiency is possible, especially in dense applications. BLE instead is built on top of the Bluetooth® protocol, which is self-reconfigurable over a series of channels inside its operative frequency range. Thus, even if it works over a usually congested frequency spectrum (since it is the same frequency also used by IEEE 802.11 standard), it is able to actively avoid collisions.

### 4.4. Costs

A generic RFID reader costs around 400$. Active RFID sensor tags sampling temperature and relative humidity can be purchased for 50$.

For what concerns Bluetooth®, an off-the-shelf or a (semi-)custom approach would provide suitable options. Bluetooth tags range around 10$ each, including a microcontroller, the radio interface and the sensors. The Bluetooth® receiver can be based on a smartphone, a PC, or a board-computer such as Raspberry Pi.

## 5. Experimental Analysis

This section carries out an experimental analysis of two systems, chosen as a representative for RFID and Bluetooth® technology. The section is divided into three subsections, two characterizing the two different architectures and one describing all the experimentally performed tests.

### 5.1. RFID Architecture

This subsection presents all the components of the chosen RFID architecture, which is composed of a *reader*, a *tag*, a *programmer* and also describes the *communication protocol*. For the purposes of this paper, it has been chosen to use a complete suite of hardware manufactured by ELA Innovation S.A (Ela Innovation S.A. https://elainnovation.com/).

#### 5.1.1. Reader

Among the possible readers, the SCIEL READER WF2 was selected for ease of connection. It is an active RFID reader operating at 433 MHz, also featuring a WiFi interface IEEE 802.11 b/g/n (2.4 GHz). It has a dedicated 9–48 V DC power supply. Its rugged enclosure ensures a wide operating temperature range: $-20{\;}^{\circ }$C to $+60{\;}^{\circ }$C. At the first start, the reader creates its own network as access point. So as to interact with other devices, the user can then connect the reader to another existing wifi network or use the one just set up. To communicate with the reader, a series of software tools are available for free download, only for Windows® platforms. The communication is practically implemented as serial over IP protocol. The software has a graphical user interface guiding the user to easily set and send the selected settings to the reader. Depending on these, the reader opens a TCP socket port on its IP address and writes accordingly the data coming from the tags. In order to collect these data, a software program has to continuously read and store them for further processing.

#### 5.1.2. Puck RHT Tag

**Figure 2:**
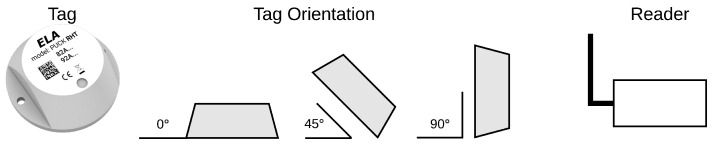
Tag Orientation.

The Puck RHT are rugged tags specifically designed for environmental monitoring applications (Puck RHT, Wireless relative humidity and temperature sensor. https://elainnovation.com/puck-rht.html). RHT stands for Relative Humidity (RH) and Temperature (T), specifying the kinds of sensors which are included on the tag. The maximum querying range is reported to be 150 m in the datasheet, in open field. The minimum interval between the queries is 200 ms, and the maximum is 10 h. The transmission of T and RH frames happens delayed only by 35 ms apart, first T and then RH. The life expectancy of the onboard non-replaceable battery strictly depends on the query rate at which it is programmed; with a 3.6 VDC coin battery it may last up to 10 years at the least frequent query rate, meaning the power consumption in this case is really low. Moreover, since the RFID tag has sensors embedded inside the enclosure, the authors reasonably expect the manufacturer designed the measurement in such a way to compensate for the possible noise due to self-heating of the device. A picture of the tag, along with the used orientations, can be found in Figure 2.

##### Relative humidity sensor

- Range: 0 to 100% RH;
- Resolution: 0.04% RH;
- Accuracy: ±2% RH max from 20% to 80%, ±5% RH max from 0 to 100%;
- Hysteresis: ±1% RH;

##### Temperature sensor

- Range: $-40{\;}^{\circ }$C to $+\;125{\;}^{\circ }$C;
- Resolution: $0.0625{\;}^{\circ }$C;
- Accuracy: $\pm 0.4{\;}^{\circ }$C max from $0{\;}^{\circ }$C to $60{\;}^{\circ }$C; $\pm 1.2{\;}^{\circ }$C for the remaining range;
- Dimensions: 57×18 mm;
- Weight 36 g.

#### 5.1.3. Programmer

The last component needed for the operation of the system is the tag programmer (SCIEL PROG IR tag programmer. https://elainnovation.com/sciel-prog-ir.html). It is not only able to program the tags for different query times, but also it can read nearby passive tags up to 15 cm distant. Furthermore, it supports batch programming for huge sets of tags and it has to be connected via USB cable to a PC running a specific program. The programmer is able to read a tag’s id and sampled data, program it, activate or deactivate it, read its software version. It also features the possibility of calibrating the temperature, i.e., insert an offset to correct its measurements.

#### 5.1.4. Communication Protocol

The query is asynchronous in the sense that the tag autonomously initiates the communication, signalling the reader that it has to transmit the data, at a time which depends on the previously set frequency. The reader then receives this kind of packet:

[AAxxxxxxLL]

where:

- **[ - ]** characters are used as delimiters;
- **AA** is a byte (2 ASCII characters) expressing signal strength;
- **xxxxxx** is the actual payload, 12 bits ID code + 12 bits measured value;
- **LL** is a byte (2 ASCII characters) defining the reader ID.

In this configuration, the whole packet transmitted is 12 bytes long.

### 5.2. Bluetooth® Architecture

In order to test the Bluetooth® Low Energy (BLE) solution, the STM32 NUCLEO low-cost prototyping system from STMicroelectronics was used. The NUCLEO hardware and software ecosystem provides an easy way to develop and evaluate embedded system applications. An STM32 NUCLEO board is equipped with an STM32 microcontroller and Arduino-compatible connectors for external peripherals, and also includes a debugger that can be connected to a PC by means of an USB cable. Then, X-NUCLEO expansion boards can be plugged on top of the base microcontroller board to add functionalities such as sensors (inertial, environmental, etc.), wireless connectivity (e.g., WiFi, BLE and LoRa®) and actuators (motor control, audio amplifier, etc.). Library drivers and code examples are available in order to accelerate and simplify development. The same architecture is used for both the sensor nodes and the data collector, and includes the NUCLEO-L152RE microcontroller board and the X-NUCLEO-IDB05A1 BLE expansion. Each sensor node is equipped also with a DHT22 relative humidity and temperature sensor, while the collector is connected to a host PC by means of a USB cable, but a WiFi expansion board might be employed to provide wireless connectivity (such as X-NUCLEO-IDW04A1).

#### 5.2.1. Microcontroller

For the current experiment, due to the limited computational demand of the application and the necessity of extending battery lifetime with the minimization of energy consumption, the NUCLEO-L152RE board was selected, which hosts the ultra-low power STM32L152RE microcontroller. Its main features are the following:

- 32-bit architecture (ARM® Cortex® -M3) clocked at up to 32 MHz
- 512 KB Flash, 64 KB RAM
- 11 peripheral communication interfaces (USB 2.0, USART, SPIs, I2Cs)
- 11 programmable timers
- 12-bit ADC and DAC
- 1.65 V to 3.6 V power supply
- customizable low-power modes, including 195 μA/MHz Run mode, 290 nA Standby mode (1.11 uA with active Real Time Clock - RTC) - at 3.3 V.

The board works in the $-40{\;}^{\circ }$C to $85{\;}^{\circ }$C temperature range and the power supply is provided either by the host PC through the USB cable, or by an external source (cable or battery): VIN (7 V–12 V), E5V (5 V) or 3.3 V power supply pins.

#### 5.2.2. Bluetooth® Low Energy Connection

The X-NUCLEO-IDB05A1 board hosts a SPBTLE-RF Bluetooth® Smart 4.1-compliant module integrating a BlueNRG-MS chip and a chip antenna. Its key features are:

- BLE Master and Slave mode supported also simultaneously
- Embedded protocol stack (GAP, GATT, SM, L2CAP, LL, RFPHY)
- Tx power: + 4 dBm
- Rx sensitivity: −88 dBm
- SPI interface, with programmable interrupt and reset
- Operating supply voltage from 1.7 to 3.6 V
- Current consumption of 1.98 uA (standby), 0.850 mA (advertising), 0.105 (connection), 7.72 mA (host in scan mode) - at 3.3 V.

The operational temperature range is between $-40{\;}^{\circ }$C and $85{\;}^{\circ }$C, and power is provided by the microcontroller board.

#### 5.2.3. DHT22

The DHT22 (DHT22 relative humidity and temperature sensor. https://www.sparkfun.com/datasheets/Sensors/Temperature/DHT22.pdf) is a low-cost temperature and relative humidity sensor. It is widely used in IoT projects thanks to its ease of use and availability of high-level code libraries. The sensor has to be supplied with 3.3 V–5.5 V voltage; the data pin needs a pull-up resistor of 1 KΩ to be functioning properly; the highest current consumption is required during the conversion of the data, and it is around 2.5 mA, while it draws 40 to 50 uA in standby; T readings range from −40 to $80{\;}^{\circ }$C, with an accuracy of $\pm 0.5{\;}^{\circ }$C; RH readings range from 0% to 100% with an accuracy of 2% to 5%; sampling frequency is around 0.5 Hz, so new data are ready every 2 s. The DHT22 sensor is connected via wire a 15 cm wire to the main board in order to avoid a significant impact of device operational heating on its measurement.

### 5.3. Tests

In order to evaluate the quality of the systems taken into consideration, a series of tests have been carried out. It has to be specified that *quality* here encompasses both the transmission efficiency and the accuracy in measurements.

#### 5.3.1. Transmission Efficiency

In order to quantify the transmission efficiency, the two systems have gone through a test in which they were put to work in several settings, changing the devices orientation and distance from the reader/receiver.

**Figure 3:**
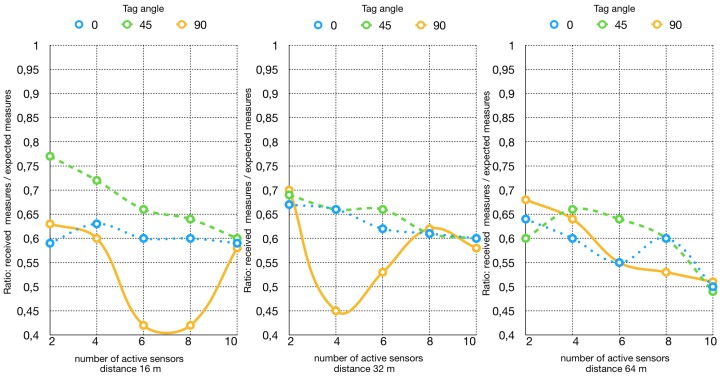
RFID reception rate taking into account tag orientation.

The tags were programmed to transmit at an interval of 1.1 s, and a time interval of 3 min was defined. In Figure 3 the result of the test is summarized. As the distance from the reader grows higher, the efficiency lowers. Increasing the number of tags used, the number of received packets subsequently increases, but the efficiency gets lower, since a higher number of packets were expected to arrive. The average efficiency for this test is in the order of 60%. It has to be pointed out that the tag orientation with respect to the reader antenna actually plays a role in data collection percentage. The $45^{\circ }$ orientation was the one to attain the highest efficiency, probably due to the reflection of the electromagnetic signals inside a closed environment. In contrast, $90^{\circ }$ orientation was the one attaining the lowest efficiency. Notwithstanding the different tag orientations, almost all the curves coincide in the right side of the graph, which means the efficiency when 10 tags were used is practically the same in every configuration.

The Bluetooth® system has been put to work in the following configuration: 6 devices in *Server mode* and one device in *Client mode*. *Server* means that this kind of devices periodically (every 1 s) send a broadcast packet, while the *Client* continuously receives and parses all the packets sent in broadcast by all the other devices communicating in the same technology. This kind of operating mode is known as *advertising* mode; no handshake is performed and no direct communication channel is established between *Server* and *Client*. The BlueNRG-MS device can be programmed both for the broadcast period and the actual broadcast message being sent. In this way, it is possible to periodically change the broadcast packet to include the new data just sampled by the sensors. In order to distinguish the packets coming only from NUCLEO boards, the broadcast package is thus composed:

<MAC_address>NUCLEO<temperature><relative_humidity>

In this way, the *Client* can actively distinguish and keep only the packets coming from the *Server*, discarding messages coming from other kinds of devices. The kept packages are forwarded via Serial communication to a PC onto which a C program is continuously reading on the port and writing to a file. Thanks to the possibility of choosing the 1 s interval, there was no need to define a time window for the test, in contrast with the RFID one. Therefore, each *Server* device was programmed to send 200 packets (running for 200 s) and then enter the sleep state. This number has been chosen in order to put the two systems in the most similar configuration, expecting almost the same total number of packets at almost the same frequency.

**Figure 4:**
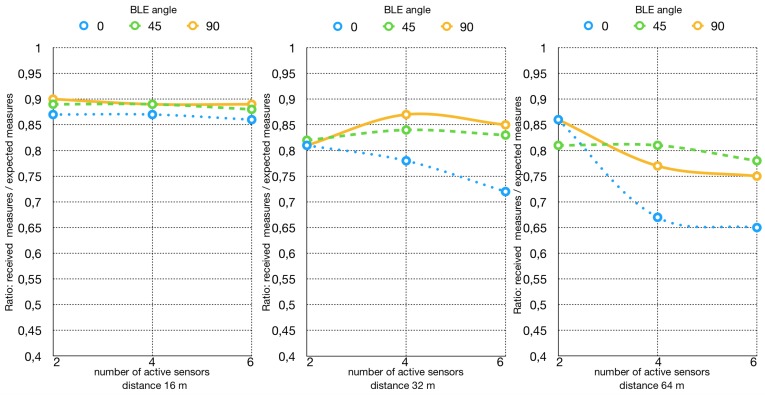
Bluetooth® reception rate taking into account tag orientation.

In Figure 4 the result of the test is summarized. At 16 m, the efficiency is almost 90% for all the three possible orientations ($0^{\circ },45^{\circ },90^{\circ }$), with no significant variation as the number of devices increases. The efficiency at 32 and 64 m can be considered stable for what concerns the $45^{\circ },90^{\circ }$ orientation as the number of devices increases. On the other hand, the orientation at $0^{\circ }$ is the one achieving the least efficiency, slightly higher than 70% at 32 m and slightly lower than 70% at 64 m. Comparing the graphs for the two systems, the reader can see that the Bluetooth® system performs better in every possible configuration. It is actually able to guarantee a higher packet reception rate at any of the considered transmission ranges, also maintaining a certain stability, in contrast with the behaviour of the RFID system, which is less stable depending on the configuration.

#### 5.3.2. Long-Range Test

To study the maximum transmission ranges of the two systems in a real scenario, a long-range test has been performed, which took place in an open area.

**Figure 5:**
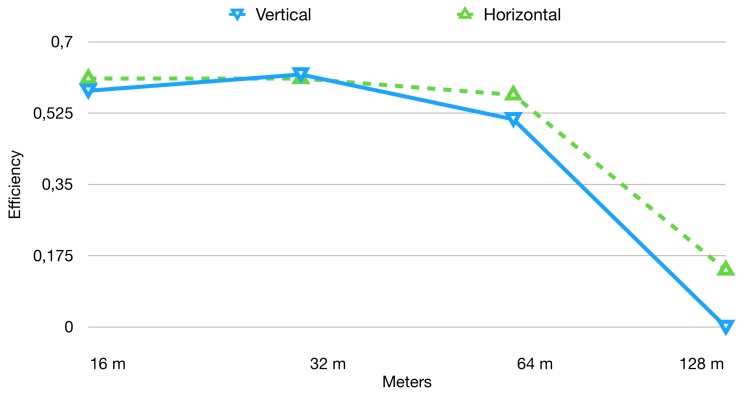
RFID data reception rate in long-range tests.

For what concerns the RFID system, 10 sensor tags have been programmed for 1.1 s query time. As in the previous experiment, the considered time window was of 3 min. The tags were placed with the top pointing right towards the reader, while the reader antenna was oriented pointing first to the sky and then in the direction of the tags. Actually, as it can be seen from Figure 5, there is not much difference between the two configurations. In one of the configurations, it is possible to observe a small increase from 16 m to 32 m. This behavior is due to the inconstant interferences generally present in real locations. Therefore, it does not mean that better transmissions are possible at 32 m, while that within a short range the distance does not affect significantly the transmission efficiency. This conclusion is also supported by the results of the indoor test, which shows small fluctuations in the efficiency level between 16 and 32 m, without an evident pick. Instead, when attempting the communication at 128 m, a dramatic loss of performance can be observed. The configuration with the reader antenna in the horizontal position was only able to attain 15% efficiency, while the one with the antenna in vertical position was actually 0%.

**Figure 6:**
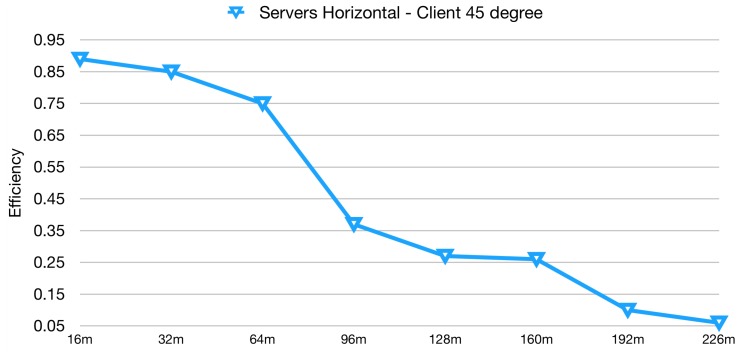
Bluetooth® data reception rate in long-range tests.

For the Bluetooth® test, the *Server* devices have been set up to for a 1 s interval between consecutive broadcast messages. As to find out the orientation of the tags ensuring the highest reception rate, several tries have been performed (antennas at $90^{\circ }$, $45^{\circ }$, $0^{\circ }$), for both kinds of devices. The resulting configuration was $45^{\circ }$ for the *Client* device (facing upwards) and $0^{\circ }$ for the *Server* devices. Figure 6 plots the results obtained for this test. As it can be inferred starting from the test at 16 m, the BlueNRG equipped devices are able to ensure a high reception rate, approximately 90%, which is 38% more with respect to the RFID case. At 128 m the reception rate was above 25%, while for the RFID system was only 17%. It was then decided to continue increasing the range at 32 m steps, until a minimum reception rate threshold was no more satisfied. This threshold was set to 5%, as minimum requirement to distinguish between effective and not effective communication. Taking this threshold as a reference, the test can be considered successful up to 226 m range, which is an interesting result compared with the 128 m reached by the RFID system.

#### 5.3.3. Accuracy in Measurements

A series of tests have been carried out to esteem measurement accuracy for temperature and relative humidity. For what concerns the RFID system, the sensor tags have been compared against the values coming from a DHT22 sensor, which was used as a reference.

The measurement accuracy test has been conducted using a refrigerator. The test lasted for several hours, during which the refrigerator has been powered on and let reach stationary conditions at a certain temperature. It has then been powered off and let heat back to room temperature. In the following subsections, 6 h of the heating phase are depicted and analyzed, for both the systems. In both cases, the sampling interval has been set at 1 s, even if given the time constant of temperature and relative humidity a sampling interval of 1 min is completely appropriate.

**Figure 7:**
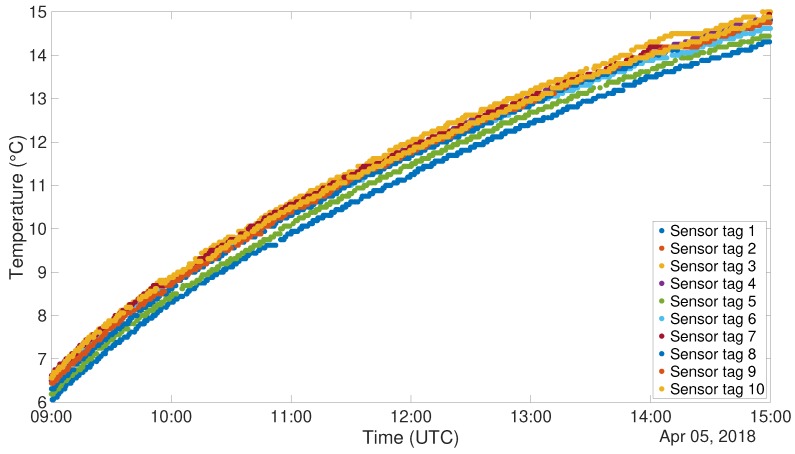
RFID Refrigerator test - Temperature (${}^{\circ }$C).

**Figure 8:**
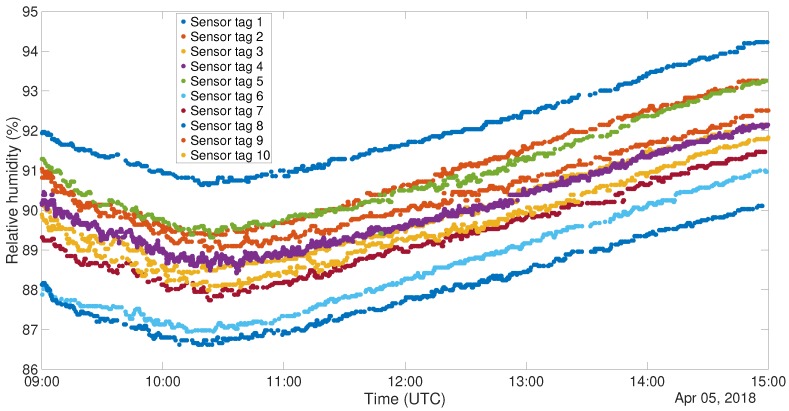
RFID Refrigerator test - Relative humidity (%).

In a test of RFID sensor tags, 10 PUCK RHT tags have been used. Both the RFID tags and one DHT22 sensor have been placed inside the cited refrigerator. Plots for temperature and relative humidity can be seen in Figure 7 and Figure 8. The plots represent the data after the application of a 5 samples window median filter. It is possible to understand, from both the figures, that the sensors embedded on the PUCK RHT tags are coherent with each other and clearly respecting the tolerance specified in the data-sheet.

To numerically quantify the correlation among the different tags, the correlation factors for every couple of sensors have been produced. Tables containing these values are presented in Table 4 and Table 5. The more the values tend to 1, the more the two sensor tags produce a similar value.

**Figure 9:**
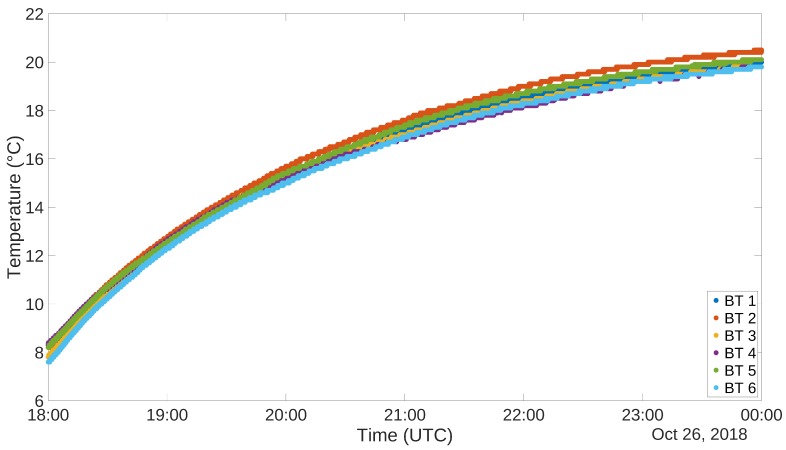
DHT22 Refrigerator test, Temperature (${}^{\circ }$C).

**Figure 10:**
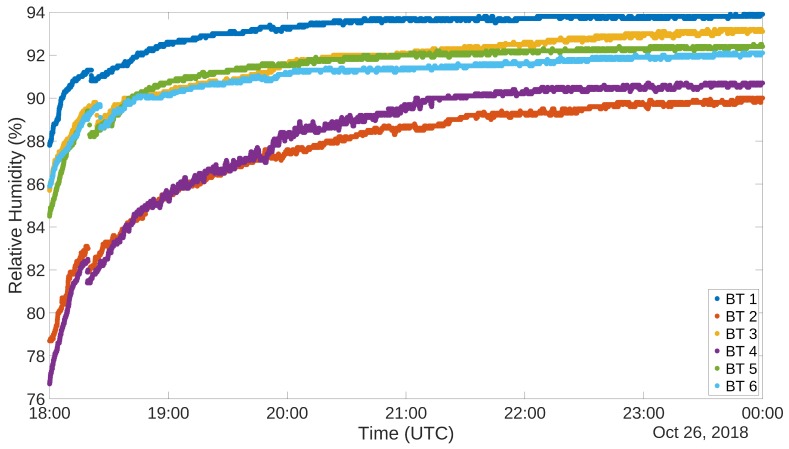
DHT22 Refrigerator test, Relative Humidity (%).

To carry out this test for the other system, 6 NUCLEO boards, equipped with BLE shield and DHT22 sensor, have been programmed to act as *Server* and placed inside the cited refrigerator. Outside of the refrigerator, another device, programmed as a *Client*, was placed in order to gather all the acquired data. Figure 9 and Figure 10 show the plots of the gathered data during the 6 h ascending phase of the refrigerator. It has to be noted though that the two experiments have been carried out in different times of the year, thus the room temperature resulted in being different. In fact, when the RFID test was performed, the air conditioning cooling system was working. This is the reason why the final temperatures for the two tests are not the same. The different setting of the experiment does not invalidate the correctness of its outputs. The graphs for the temperatures are definitely well aligned, and this can also be said for what concerns relative humidity, taking into account the usual uncertainty in measuring this kind of environmental data. Also in this case correlation factors for sensor couples have been produced. These data are organized in distance Table 6 and Table 7. Also in this case correlation values are extremely high, confirming the correspondence among the different instances of the sensor.

**Figure 11:**
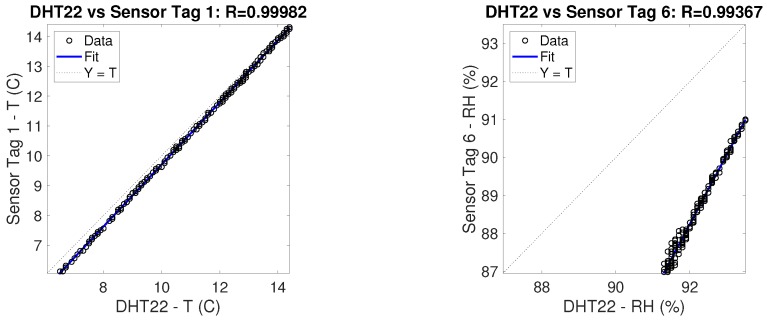
Correlation between DHT and Sensor tag sensors - Temperature and Relative Humidity.

Having used the DHT22 sensor as a reference, a comparison between the measurements of the sensor itself and of the sensor tags is due. As previously done among instances of RFID sensor tags and DHT22, correlation factors and correlation graphs have been produced comparing the measurements of the two kinds of devices. These graphs are represented in Figure 11. From these graphs it is possible to infer the high correlation between the devices so as to support the suitability of the use of this kind of RFID sensor tags for the purpose of indoor thermal monitoring. For both graphs in Figure 11, a little shift of values can be appreciated due to the physical construction of the specific couple of sensors. The absolute value of the shift is nevertheless comprised inside the tolerance limits given by the datasheets (maximum ±5% for both DHT22 and RFID sensor tag). In order to further reduce such shift a specific calibration would be required, but for this kind of application it would represent an additional cost for each sensor that would eventually be too expensive.

#### 5.3.4. Point to Point Calibration

To further prove the accuracy of the used devices and their applicability for the purpose of this paper, their sampled values have been compared against the ones produced by a professional sensing device. The chosen sensor is the HygroPalm *HP22* by Rotronic®, with a HygroClip2 HC2-S sensing probe. This probe has an accuracy of ±0.8% for relative humidity and ±0.1 K for temperature.

**Figure 12:**
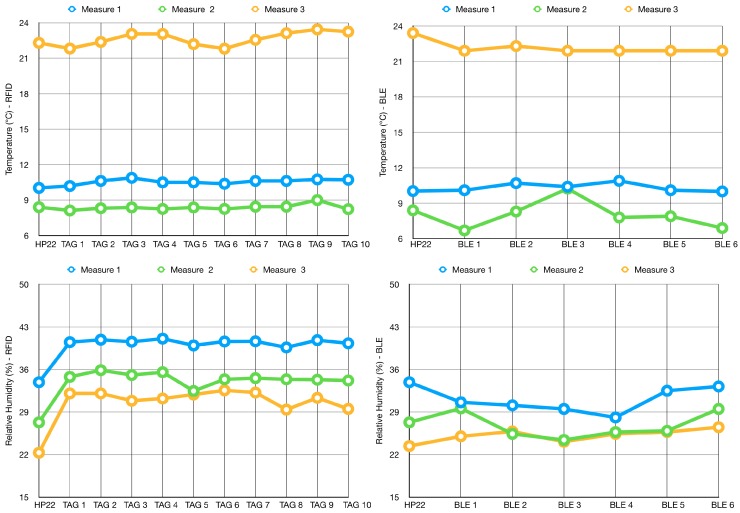
Point to point calibration test.

The test was executed in three different runs in three different conditions for what concerns temperature and relative humidity conditions. The graphs in Figure 12 show the plots of the three tests. Graphs on the top concern measurements of temperature, while the ones at the bottom concern measurements of relative humidity. Graphs on the left represent data gathered by the RFID sensor tags, while those on the right represent data gathered by DHT22 sensors connected to Bluetooth®modules. For all the graphs, the reference measurement gathered by the HP22 sensor is the first value on the left. As already pointed out in the previous subsection, temperature data are well aligned, while the relative humidity ones clearly show an offset. Nevertheless, they are also characterized by a strong correlation.

#### 5.3.5. Power Consumption Estimation

Some reasoning has to be performed for what concerns the power consumption of the NUCLEO BLE devices. Let us logically split the consumption into two parts:

- microcontroller with sensor
- BLE module

**Figure 13:**
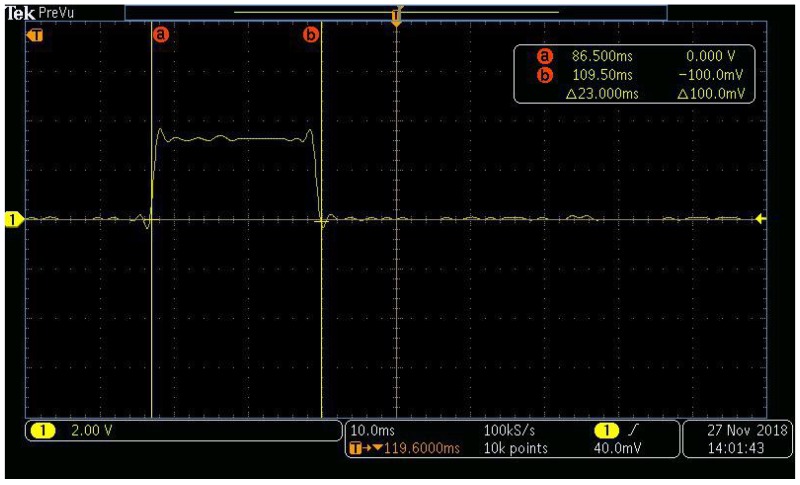
Microcontroller active time.

Once every minute, the microcontroller reads the sensor and programs the BLE module to broadcast the acquired temperature and humidity data. As it can be seen from Figure 13, this process takes around 23 ms. This has been achieved setting a GPIO pin to high value only during the active phase. The current consumed by the microcrontroller and the sensor in this active phase is around 12 mA. During the off phase, the microcontroller current is negligible, while the DHT22 sensor instead keeps draining a significative amount of current. The average current consumed by these modules in the off phase is around 45 μA.

**Figure 14:**
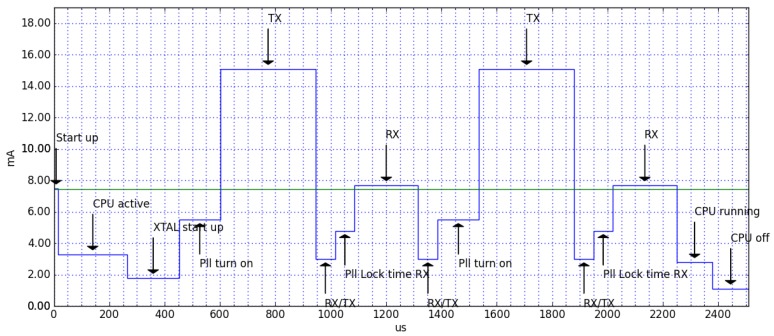
BLE current consumption plot.

The BLE module then uses a single advertising message sent on two channels for transmitting the data to the receiver. After this, the whole system enters a low-power mode. Figure 14 is an excerpt of the BlueNRG current consumption estimation tool (https://www.st.com/en/embedded-software/stsw-bnrg001.html). This figure shows the current consumed only by the BLE shield to transmit a 27-byte packet over 2 channels at the maximum power of 7 dB. This operation lasts for 2.5 ms, during which the device consumes 7.4 mA. During the rest of the time, it is in low power mode, consuming only 1.7 μA.

In this configuration, a 1000 mAh coin battery (such as the CR2477) could last for more than 2 years. It has to be noted that the highest consumption is given by the DHT22 sensor in sleep mode for the majority of the time. Possible solutions to overcome this useless current drain could be: the use of another kind of sensor; inserting a transistor in the design of the custom design in order to totally cut off the power to the sensor when it is not required.

### 5.4. Case Study

**Figure 15:**
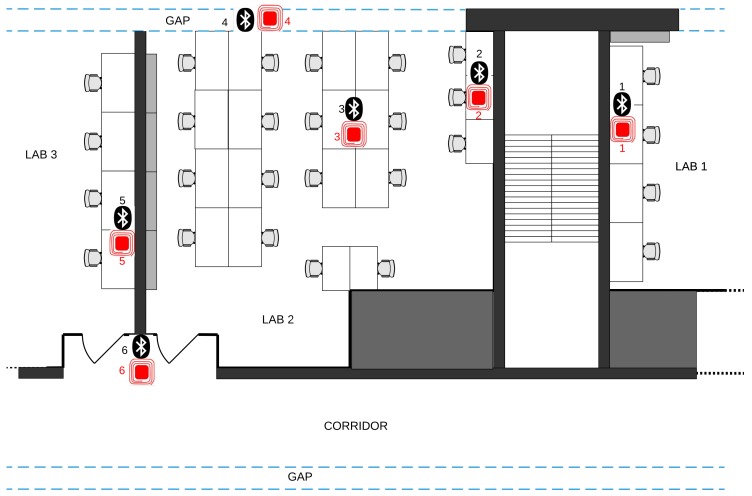
Case study plant.

The case study test has been carried out having the two different systems sampling together at the same time in the same location for every couple (one RFID device and one Bluetooth® device). This test took place inside the Department of Control and Computer Engineering at Politecnico di Torino, Italy. Figure 15 shows the map of the sensed area, with the deployment locations of the couples of sensors. In total, 6 couples of sensors have been deployed: 6 RFID and 6 Bluetooth® devices. Two couples of sensors have been placed inside Laboratory room number 2, while the other couples have been deployed in the surrounding areas. The RFID receiver and the NUCLEO *Client* device have been placed in Laboratory 2, near the deployment location of devices #3. All laboratories in the map do not have direct exposure on the exterior of the building, on one side there is a corridor, while on the other there is a gap between window glasses which serves as an insulator in case the outside windows are open. During the tests, windows on the outside were open, meaning that temperature and relative humidity values were actually the open air ones.

**Figure 16:**
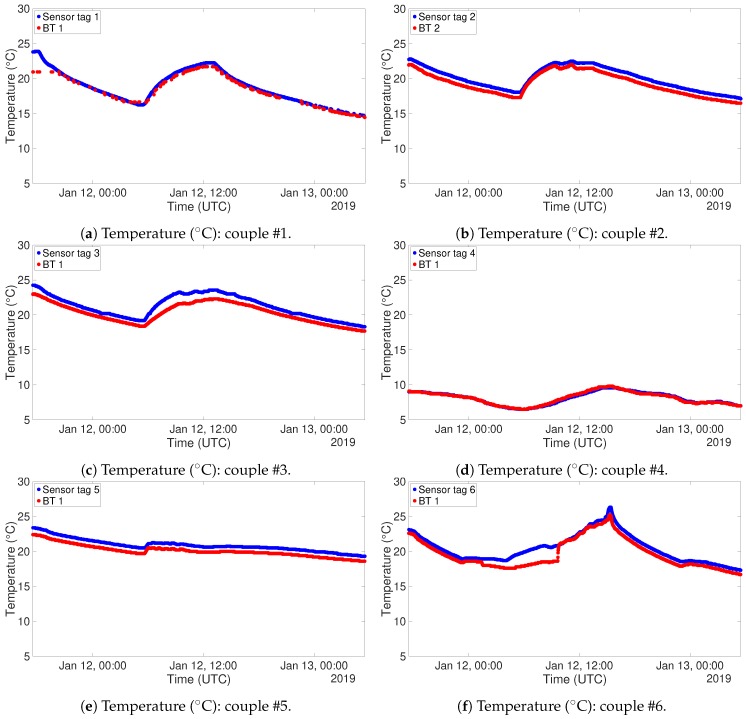
Temperature (${}^{\circ }$C) measurements: RFID (blue) and BT (red).

**Figure 17:**
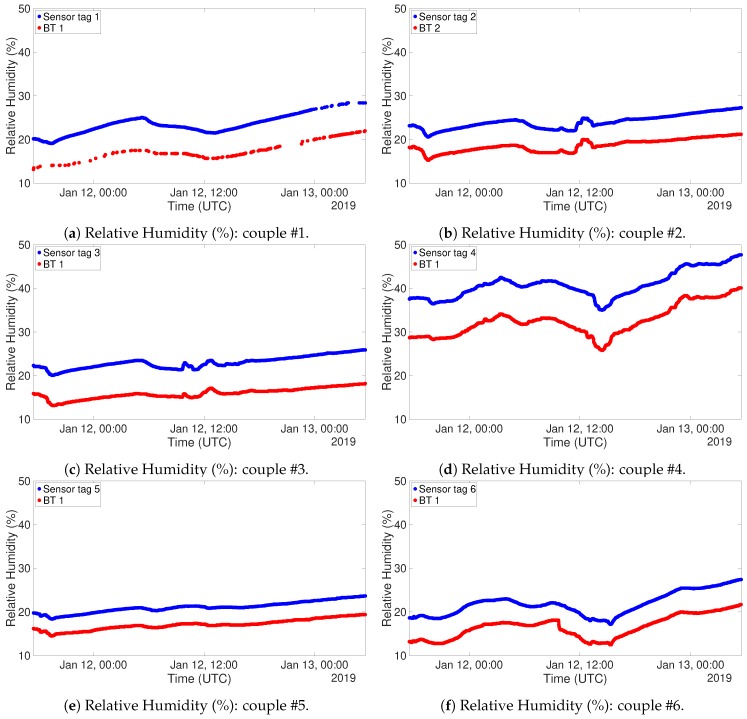
Relative Humidity (%) measurements: RFID (blue) and BT (red).

For this test, the devices have been programmed for a sampling interval of one minute, since it is reasonable to expect that possible variations are not instantaneous. The gathered data are presented in Figure 16 for what concerns temperature, and in Figure 17 for what concerns relative humidity. These graphs have been organized in such a way to highlight the behaviour of couples of sensors. Data coming from RFID devices have been plotted in blue, while those coming from Bluetooth® devices have been plotted in red. In this way, the comparison between the two measurements is straightforward. The continuous curves show that the system has been able to receive a congruous amount of data, despite walls and interference coming from other sources of electromagnetic signals. The only couple of devices which suffered slightly more from this point of view was the first one, due to the fact was the farthest from the reader devices and the signal had to pass through several walls and metallic stairs. The alignment for both temperature and relative humidity is clear from both the figures. The one related to relative humidity actually displays the couples of sensing devices with an offset among the two, but their evolution throughout time is the same. As previously said, this kind of offset could be corrected with a calibration of the devices. Nevertheless, the sensors measured fulfilling their accuracy ranges. It has to be noted that the couple of devices #6 has been placed in such a place that there was sunlight hitting directly the devices. This fact has a direct impact on the temperature sensing, as it can be inferred by the peak registered on Jan 12th around 16 PM.

## 6. Conclusions

In this paper, an investigation on wireless technologies applied to the task of thermal monitoring is proposed. This task falls into the IoT scenario, in which the measurement of indoor temperature and relative humidity are demanded to autonomous devices able to control the HVAC system in such a way to reduce power consumption for the related appliances. Wireless technologies have been chosen due to their ease of deployment and scalability with respect to wired ones. This study focused on two different systems, a commercial one and a semi-custom one. The former exploiting RFID technology, while the latter exploiting BLE technology. The two systems have been carefully compared taking into account several key requirements that must be fulfilled to guarantee the correctness and the dependability of the overall apparatus. The two technologies have been first described and analyzed from a theoretical point of view, generically considering their operating range, scalability, interference resilience and costs of deployment. The two actually chosen systems have then been described and a comparison between them has been performed through the analysis of the outcome of a series of tests. These tests showed that both systems are able to sample the needed data with an uncertainty not invalidating the correctness of the measurement. For what concerns the transmission range and data reception ratio, the BLE system usually performs better in both the tasks. A final case study has been carried out, with comparable outcomes for both the technologies. The main difference resides in the scalability of the system. The RFID one, once reached the maximum transmission range, requires the deployment of an additional reader. The BLE one instead could be set to form a mesh network, but this configuration has the downside of the increased power requirements. Concluding, there is no prominent technology to choose for what concerns the task of thermal monitoring, a trade-off has to be carefully performed case by case. For example, if the final network configuration is not expected to change, probably an RFID system could be preferred, otherwise, in a dynamic environment, the reconfigurability of the BLE comes in handy.

## Figures and Tables

**Table 1:** RFID Frequencies.

	Frequency	Range	Active/Passive
Low Frequency (LF)	125–134 KHz	1–10 cm	Passive
High Frequency (HF)	13.56 MHz	10 cm–1 m	Passive
Ultra High Frequency (UHF)	433 MHz	1–100 m	Active
	866–868 MHz in EU,	1–12 m	Passive
	902–928 MHz in USA		
Microwave	2.45–5.8 GHz	1–2 m	Active
	3.1–10 GHz in USA	Up to 200 m	

**Table 2:** BLE specifications: GAP roles.

GAP Role	Description
BROADCASTER	A device that only sends advertising events
OBSERVER	A device that only receives advertising events
PERIPHERAL	A device that accepts the establishment of an LE
	physical link using the connection establishment procedure
CENTRAL	A device that supports the Central role initiates the
	establishment of a physical connection

**Table 3:** RFID and Bluetooth®  comparison table.

	RFID	Bluetooth®
Range	Medium	High
Scalability	Medium	High
Interference resilience	Medium	High
Cost	Low	Medium
Power consumption	Low	Medium

**Table 4:** Correlation among different instances of RFID tags - Temperature (${}^{\circ }$C).

	Tag 1	Tag 2	Tag 3	Tag 4	Tag 5	Tag 6	Tag 7	Tag 8	Tag 9
**Tag 2**	0.99974								
**Tag 3**	0.99976	0.99982							
**Tag 4**	0.99979	0.99979	0.99964						
**Tag 5**	0.99985	0.99991	0.9998	0.99978					
**Tag 6**	0.99985	0.99978	0.99955	0.99982	0.99986				
**Tag 7**	0.99951	0.99969	0.99986	0.99959	0.99972	0.99961			
**Tag 8**	0.99986	0.9998	0.99982	0.99975	0.99979	0.99985	0.99968		
**Tag 9**	0.99973	0.99975	0.99985	0.99973	0.99985	0.99983	0.9997	0.99961	
**Tag 10**	0.99972	0.99976	0.99958	0.99975	0.99972	0.99982	0.99947	0.99988	0.99966

**Table 5:** Correlation among different instances of RFID tags - Relative Humidity (%).

	Tag 1	Tag 2	Tag 3	Tag 4	Tag 5	Tag 6	Tag 7	Tag 8	Tag 9
**Tag 2**	0.99448								
**Tag 3**	0.99853	0.99513							
**Tag 4**	0.9925	0.98212	0.99273						
**Tag 5**	0.98851	0.98609	0.99606	0.99162					
**Tag 6**	0.9957	0.99676	0.99838	0.96458	0.98923				
**Tag 7**	0.99547	0.99694	0.9971	0.99035	0.99011	0.99689			
**Tag 8**	0.99583	0.99803	0.99623	0.98767	0.98235	0.98543	0.99258		
**Tag 9**	0.98334	0.92486	0.99371	0.99169	0.9886	0.98401	0.99578	0.96842	
**Tag 10**	0.99684	0.98727	0.98511	0.99276	0.98118	0.97046	0.99631	0.99179	0.98741

**Table 6:** Correlation between different instances of DHT22 - Temperature sensor.

	Sens. 1	Sens. 2	Sens. 3	Sens. 4	Sens. 5
**Sens. 2**	0.99982				
**Sens. 3**	0.99913	0.99955			
**Sens. 4**	0.99917	0.99941	0.99953		
**Sens. 5**	0.99987	0.99984	0.99923	0.99911	
**Sens. 6**	0.99948	0.99976	0.99983	0.99965	0.99955

**Table 7:** Correlation between different instances of DHT22 - Relative Humidity sensor.

	Sens. 1	Sens. 2	Sens. 3	Sens. 4	Sens. 5
**Sens. 2**	0.97215				
**Sens. 3**	0.95111	0.9876			
**Sens. 4**	0.97429	0.99622	0.9858		
**Sens. 5**	0.99417	0.97092	0.95288	0.97182	
**Sens. 6**	0.97742	0.98093	0.98119	0.98095	0.98258

## Abbreviations

The following abbreviations are used in this manuscript:

RFID	Radio-frequency identification
IoT	Internet of Things
HVAC	Heating, Ventilation and Air Conditioning
BLE	Bluetooth® Low Energy
BlueNRG	Bluetooth® Low Energy
